# Revision Surgery in Permanent Patellar Dislocation in DiGeorge Syndrome

**DOI:** 10.1155/2015/752736

**Published:** 2015-12-13

**Authors:** Massimo Berruto, Andrea Parente, Paolo Ferrua, Stefano Pasqualotto, Francesco Uboldi, Eva Usellini

**Affiliations:** SSD Chirurgia Articolare del Ginocchio, Istituto Ortopedico Gaetano Pini, Piazza Cardinale Andrea Ferrari 1, 20122 Milano, Italy

## Abstract

A 29-year-old patient, suffering from DiGeorge syndrome, came to our attention with a history of persistent pain and patellar instability in the left knee after failure of arthroscopic lateral release and Elmslie-Trillat procedure. The patient was unable to walk without crutches and severely limited in daily living activities. Because of arthritic changes of the patellofemoral joint and the failure of previous surgeries it was decided to perform only an open lateral release and medial patellofemoral ligament (MPFL) reconstruction using a biosynthetic ligament in order to obtain patellofemoral stability. At one year post-op range of motion (ROM) was 0–120 with a firm end point at medial patellar mobilization; patella was stable throughout the entire ROM. All the scores improved and she could be able to perform daily activity without sensation of instability. Bilateral patellar subluxation and systemic hyperlaxity are characteristics of syndromic patients and according to literature can be also present in DiGeorge syndrome. MPFL reconstruction with lateral release was demonstrated to be the correct solution in the treatment of patellar instability in this complex case. The choice of an artificial ligament to reconstruct the MPFL was useful in this specific patient with important tissue laxity due to her congenital syndrome.

## 1. Introduction

DiGeorge syndrome is a very rare genetic condition characterized by a 22q11 micro deletion clinically resulting in cardiac abnormality, abnormal faces, thymic aplasia, cleft palate, and hypocalcemia/hypoparathyroidism [1].

According to literature one of the pathological traits of this syndrome could be objective patellar instability [2]. The main issue in treating this complex case of patellofemoral instability is considering not only knee's altered biomechanics but also patient's general condition. In this case we chose, according to our experience in patellofemoral instability treatment and poor patient's compliance, to avoid aggressive surgery. In order to restore the proper patellofemoral biomechanics by mini-invasive treatment, an open lateral release and MPFL reconstruction with a biosynthetic ligament were performed.

## 2. Case Presentation

In May 2013 a 29-year-old female patient, suffering from DiGeorge syndrome, came to our attention with a history of bilateral patellar pain, instability, and limping since the age of six. In 2005 the patient underwent an arthroscopic lateral release [3] and an Elmslie-Trillat procedure [4] in her left knee with no improvement neither in pain nor in patellar instability. In 2008 she underwent a revision surgery in the left knee: proximal and distal patellar realignment and treatment of a patellar cartilage lesion with autologous chondrocyte implantation (ACI) technique were performed [5].

After a couple of months the patient complained about recurrence of pain and instability.

The use of a wheel chair was necessary to cover great distance and at home she was unable to walk without crutches.

In May 2013 at the clinical examination the knee showed no effusion; it was slightly valgus with a ROM of 5–130°. The patellar mobility was 4/4 [6] with a positive apprehension test and medial patellofemoral ligament (MPFL) was insufficient. Laxity score according to Beighton criteria was 6. Patella was permanently luxated in flexion and palpation of patellar facets caused severe pain. IKDC subjective score was 8, KOOS 10.7, KUJALA 9, VAS 7, and TEGNER 0 [7–9].

**Figure 1:**
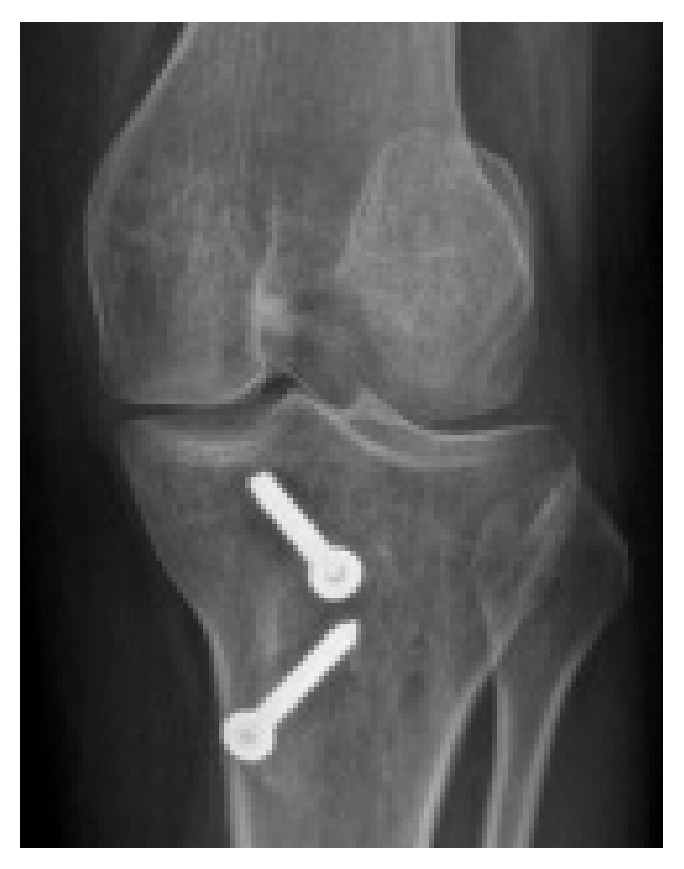
Pre-op AP knee X-ray.

**Figure 2:**
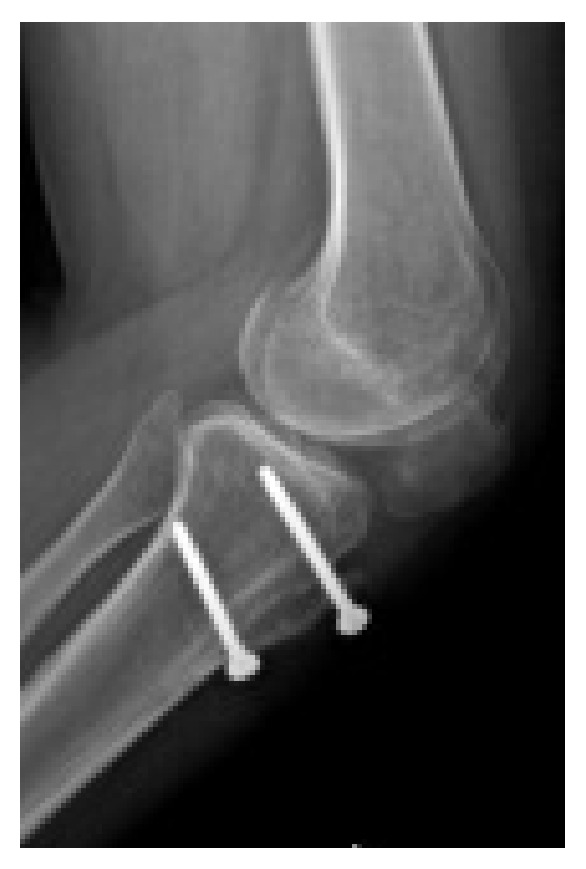
Pre-op lateral knee X-ray.

**Figure 3:**
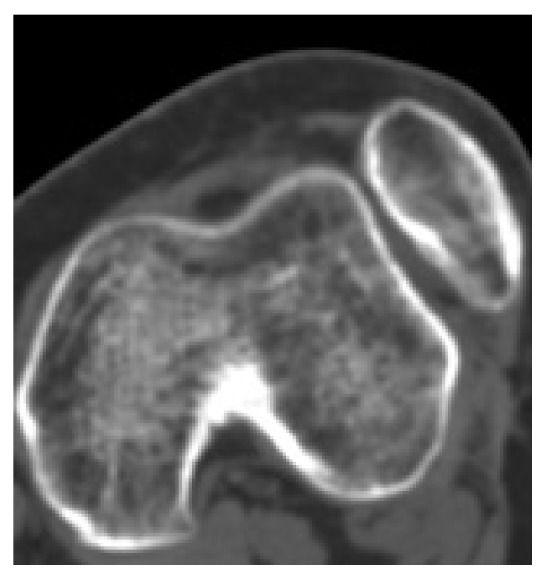
Pre-op knee CT demonstrating patella dislocation.

Preoperative imaging (traditional X-rays and CT scan) showed grade C trochlear dysplasia [10] and a permanent lateral dislocation of the patella (Figures 1 and 2) with a pseudo-patella infera (Caton-Deschamps index = 0.6) [11]; CT imaging described a tibial tubercle to the trochlear groove (TT-TG) distance of 25.9 mm and a patellar tilt of 62° (Figures 1–3).

A one-step diagnostic arthroscopy, hardware removal, open lateral release, and MPFL reconstruction with a biosynthetic ligament (LARS R6 X 400, LARS, Arc sur Tille, France) were scheduled [12].

**Figure 4:**
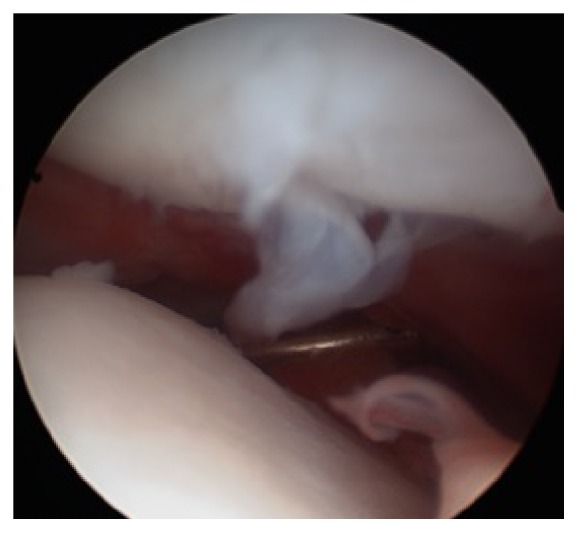
Cartilaginous flap of the patella in arthroscopic view.

The arthroscopic examination showed a grade 2 International Cartilage Repair Society (ICRS) scale [5] chondral lesion of the patella in the bearing zone, where ACI was performed. It was decided not to treat the lesion (Figure 4).

**Figure 5:**
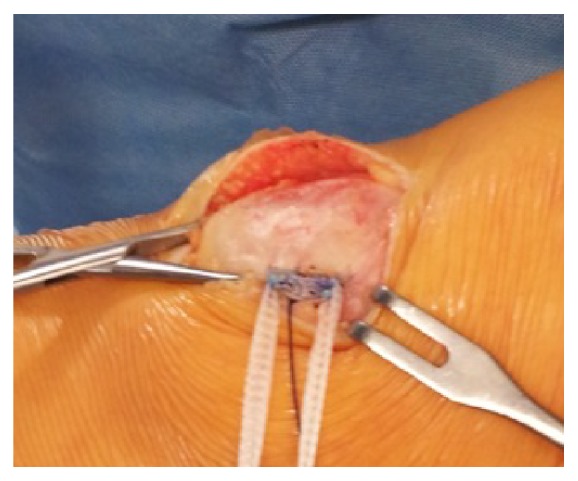
LARS anchored to the patella in MPFL reconstruction. Intraoperative view.

After a standard open lateral release procedure [13] the patella was reducible in the trochlear groove also in flexion. MPFL reconstruction was performed through a medial parapatellar incision: the biosynthetic graft was fixed on the medial patellar ridge using two Juggerknot tissue anchors (Zimmer Biomet, Warsaw, IN, USA) (Figure 5). Femoral half tunnel (7 mm wide and 40 mm long) was performed after identifying the correct insertion point according to Schottle et al. [14]. The isometry of the graft was checked all throughout the range of motion and the fixation was performed at 30° of flexion with a Biorci bioresorbable screw (S&N, Andover, MA, USA) [15–17].

A final check showed a 2/4 medial-lateral mobility, a firm end point feeling at lateralization, and a stable patella all throughout the range of motion.

Partial weight bearing with crutches was immediately allowed with knee braced in extension and progressive passive motion recovery with CPM started increasing until 110° of flexion for the first month. Intensive isometric quadriceps strengthening and hamstrings stretching were also encouraged from the very beginning of the postoperative period [17]. At one month post-op patient began closed kinetic chain exercises, proprioceptive training, and hydrobike and core stability exercises.

Clinical evaluation was performed at one, three, six, and twelve months. Function was assessed using IKDC, KOOS, and KUJALA clinical scores. Pain was evaluated with VAS scale and level of activity by TEGNER scale.

At six-month follow-up evaluation the knee showed slight effusion, a ROM of 0–120, slight pain at the palpation of all compartments and patellar facets, and firm end point at medial patellar mobilization with 2/4 mobility.

IKDC was 71.7, KOOS 76.4, KUJALA 69.2, VAS 2, and TEGNER 2.

**Figure 6:**
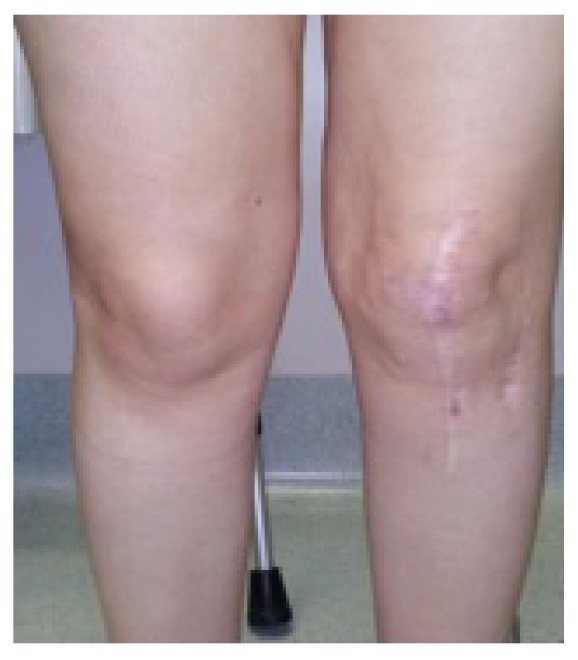
Standing view with knee extended and reduction of patella luxation at one-year follow-up.

At one-year follow-up, no effusion was noticed, the ROM was complete, and the patient had no pain at palpation. Patella was stable with negativization of all clinical tests and she could be able to perform daily activities without sensation of instability and returned to a normal life (Figure 6).

IKDC was 89, KOOS 92.2, KUJALA 82.4, VAS 0, and TEGNER 4.

An overall improvement was observed in all scores when compared to preoperative evaluation.

**Figure 7:**
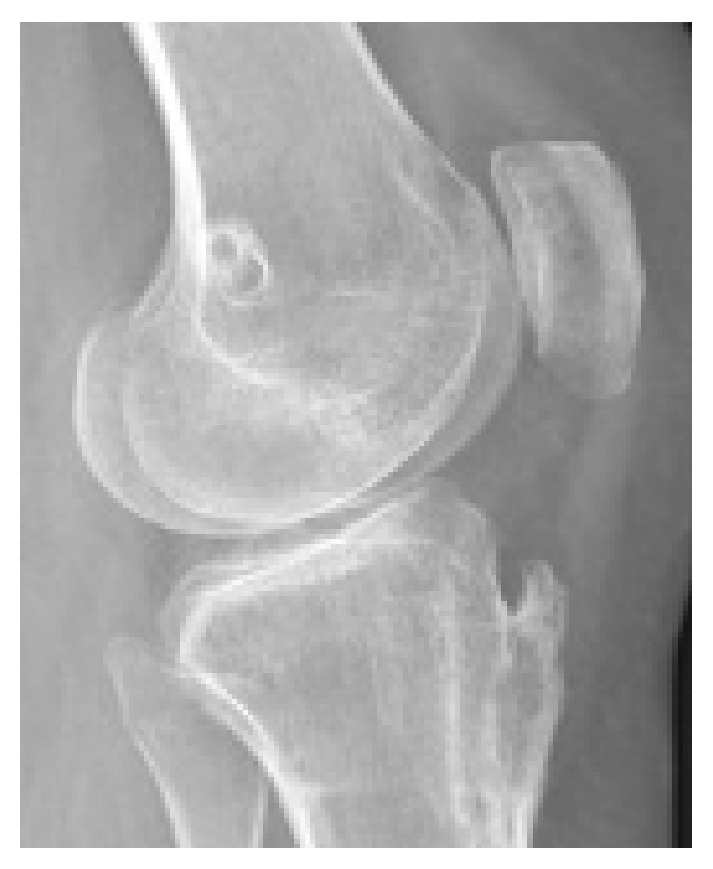
Post-op lateral knee X-ray.

**Figure 8:**
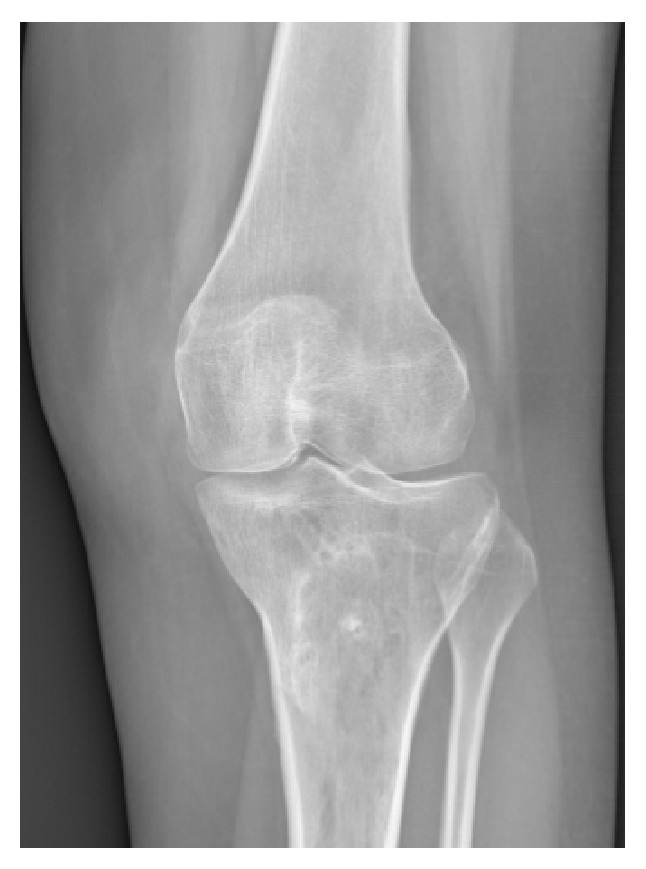
Post-op anteroposterior knee X-ray.

Post-op X-rays demonstrated the correct positioning of the neosynthetic ligament in the femur, as illustrated by Schottle et al. [14], and the reduction of the patella in the trochlea (Figures 7 and 8).

## 3. Discussion

DiGeorge syndrome has an autosomal dominant inheritance pattern caused by a 22q11 deletion and it is the most common micro deletion syndrome characterized by cardiac abnormality, such as tetralogy of Fallot, abnormal faces, thymic aplasia, cleft palate, and hypocalcemia/hypoparathyroidism.

In literature there are only two cases of patients suffering from DiGeorge syndrome and bilateral recurrent patellar subluxation in association with camptodactyly [2]. Even if these examples could not be significant, patellar instability could be a characteristic of this genetic syndrome like many others (Down, Ehlers Danlos, Marfan, etc.) and this patient should be the third case of this particular series. Given these considerations, this is the first reported case of revision surgery in patellofemoral instability in DiGeorge syndrome.

MPFL reconstruction is one of the most reliable options in surgical treatment of patellofemoral instability [18]. Several anatomical factors can predispose to this condition such as increased TT-TG, patella alta, and trochlear dysplasia; the laxity of the MPFL, major passive restraint in patella lateral translation, plays also an important role [19]. Surgical explorations found MPFL injuries in most cases of patella dislocation; indeed reconstruction of MPFL is now performed alone or associated with other procedures such as distal realignments or trochleoplasty in most of the patellofemoral instability surgical treatment series [20].

In this case the patient had undergone several surgeries to stabilize the patella, arthroscopic lateral release and the proximal and distal patellar realignment, with poor results. Patient experienced persistence of anterior knee pain and patella luxation. Moreover TT-TG was always pathological (25.9 mm), it was decided not to treat the dysplastic trochlea, grade C, in previous surgeries, and patella was persistently infera with a Caton-Deschamps index of 0.6, even though, in presence of a fixed luxation of patella, this index can be altered and it would be more correct to speak about pseudo-patella infera.

Pre-op X-rays demonstrated patellofemoral osteoarthritis grade 1 according to Iwano and the patient was not able to walk because of pain resulting in a great limitation in daily activities.

In this situation it was very important to find the best solution to solve patellofemoral pathological biomechanics and anterior knee pain and to give the patient a good quality of life, trying to be as conservative as possible, also in relation to the general condition of the patient (DiGeorge syndrome).

Thus MPFL reconstruction with the improvement of patellar tilt by lateral release in open surgery was considered the best treatment option in this case. Indeed in a situation of knee osteoarthritis it was decided not to correct TT-TG and not to perform a trochleoplasty (moreover seldom indicated in type C dysplasia) sparing the patient the need of an aggressive bony procedure, also considering the patient's poor compliance. In MPFL reconstruction a biosynthetic graft was chosen in order to prevent recurrence of instability caused by syndrome's related tissue hyperlaxity [17]. After surgery the patient experienced good quality of life, with no anterior knee pain or episode of patellar dislocation; she returned to walk in her everyday life with an important improvement in relation to the preoperative condition.
